# New record of the pentatomine stink bug species *Caystrusorientalis* Zhang and Lin (Hemiptera: Pentatomidae) from Japan

**DOI:** 10.3897/BDJ.6.e24439

**Published:** 2018-04-24

**Authors:** Haruka Tachi, Tadashi Ishikawa

**Affiliations:** 1 Laboratory of Entomology, Faculty of Agriculture, Tokyo University of Agriculture, Atsugi-shi, Kanagawa, Japan Laboratory of Entomology, Faculty of Agriculture, Tokyo University of Agriculture Atsugi-shi, Kanagawa Japan

**Keywords:** Caystrini, *Caystrusorientalis*, Heteroptera, Japan, Kyushu, Pentatomidae, Pentatomini, Ryukyu Islands

## Abstract

**Background:**

To date, the stink bug species *Caystrusorientalis* Zhang and Lin, 1985, was known to occur only in China.

**New information:**

A species known as “*Caystrus* sp.” and “*Neodinus* sp.” in Japan has been identified as *C.orientalis*, which has been recorded from Japan for the first time.

## Introduction

The stink bug species *Caystrusorientalis* Zhang and Lin, 1985, belonging to the pentatomine tribe Caystrini, was described based on one female in Fujian Province, China (Zhang and Lin 1985). Subsequently, Lin and Zhang (1993) and Lin et al. (1999) recorded it from Jiangxi and Yunnan Provinces, China. To date, the species has been known to occur in south-western and south-eastern China.

In Japan, two species of the genus *Caystrus* Stål, *C.depressus* (Ellenrieder, 1862) and *C.obscurus* (Distant, 1901), have been recorded by Takara (1957) and Ishikawa et al. (2012), respectively. However, the existence of an additional species of this genus or an allied genus has been suggested under indeterminate conditions, such as *Caystrus* sp. (Nozaki et al. 2016) and *Neodius* sp. (Nagasaki Prefecture 2001); *Neodius* Bergroth is currently regarded as a synonym of *Caystrus* (Rider 2006). Our recent closer examination of the undetermined species unequivocally verified its identity as *C.orientalis*.

The present paper reports *Caystrusorientalis* as the third representative of *Caystrus* in Japan, providing diagnostic morphological characters with photographic images to aid in accurate identification; the habit and habitat preference are also briefly discussed.

## Materials and methods

Dried specimens were used. Morphological observations, including those of genital structures, were made under Olympus SZ60 and SZX16 binocular microscopes. Digital images of the specimens were captured using a Nikon D200 digital camera body and Nikon AF Micro-Nikkor 60 mm f/2.8D lens and using a KEYENCE digital microscope system (VHX-1000 digital microscope, VHX-S50 observation system, VHX-1100 multi-scan camera and VH-Z20R zoom lens). The terminology used herein generally follows that of Tsai et al. (2011). The specimens examined in this study are preserved in the Laboratory of Entomology, Faculty of Agriculture, Tokyo University of Agriculture, Japan, except for three specimens collected from Nagasaki Prefecture, which will be housed in Nagasaki North High School, Science Club, Nagasaki City, Japan.

## Taxon treatments

### Caystrus orientalis

Zhang and Lin, 1985

3E9023A7-0A3D-55FA-ACCF-3405114029B5

Caystrus orientalis : *Zhang and Lin 1985*: 21, new species, description and figures; Lin and Zhang 1993: 125, record, description and figure; Lin et al. 1999: 70, record, description and figure; Rider 2006: 292, catalogue; Aukema et al. 2013: 456, catalogue.
Neodius sp.: Nagasaki Prefecture 2001: 428, distribution and biology.
Caystrus sp.: Nozaki et al. 2016: 93, list, description, distribution and biology.

#### Materials

- **Type status:** Other material. **Occurrence:** catalogNumber: 2017-00018; recordedBy: Tatsuya Nozaki; individualCount: 1; sex: male; lifeStage: adult; **Taxon:** scientificName: Caystrusorientalis Zhang and Lin, 1985; namePublishedIn: 1985; kingdom: Animalia; phylum: Arthropoda; class: Insecta; order: Hemiptera; family: Pentatomidae; genus: Caystrus; specificEpithet: orientalis; scientificNameAuthorship: Zhang and Lin; **Location:** islandGroup: Amakusa Islands; island: Shimo-shima Island; country: Japan; stateProvince: Kumamoto; municipality: Amakusa City; locality: Oniki-machi, Oniki; decimalLatitude: 32.233; decimalLongitude: 130.015; geodeticDatum: WGS84; **Identification:** identifiedBy: Haruka Tachi and Tadashi Ishikawa; dateIdentified: 2017; **Event:** samplingProtocol: none specified; eventDate: 2015-10-17; **Record Level:** institutionCode: ELTUA; collectionCode: IC; basisOfRecord: PreservedSpecimen
- **Type status:** Other material. **Occurrence:** catalogNumber: 2017-00019; recordedBy: Tatsuya Nozaki; individualCount: 1; sex: male; lifeStage: adult; **Taxon:** scientificName: Caystrusorientalis Zhang and Lin, 1985; namePublishedIn: 1985; kingdom: Animalia; phylum: Arthropoda; class: Insecta; order: Hemiptera; family: Pentatomidae; genus: Caystrus; specificEpithet: orientalis; scientificNameAuthorship: Zhang and Lin; **Location:** country: Japan; stateProvince: Nagasaki; municipality: Nagasaki City; locality: Mieda; decimalLatitude: 32.82; decimalLongitude: 129.73; geodeticDatum: WGS84; **Identification:** identifiedBy: Haruka Tachi and Tadashi Ishikawa; dateIdentified: 2017; **Event:** samplingProtocol: none specified; eventDate: 2016-09-16; **Record Level:** institutionCode: ELTUA; collectionCode: IC; basisOfRecord: PreservedSpecimen
- **Type status:** Other material. **Occurrence:** catalogNumber: 2017-00020; recordedBy: Tatsuya Nozaki; individualCount: 1; sex: male; lifeStage: adult; **Taxon:** scientificName: Caystrusorientalis Zhang and Lin, 1985; namePublishedIn: 1985; kingdom: Animalia; phylum: Arthropoda; class: Insecta; order: Hemiptera; family: Pentatomidae; genus: Caystrus; specificEpithet: orientalis; scientificNameAuthorship: Zhang and Lin; **Location:** islandGroup: Amakusa Islands; island: Shimo-shima Island; country: Japan; stateProvince: Kumamoto; municipality: Amakusa City; locality: Oniki-machi, Oniki; decimalLatitude: 32.233; decimalLongitude: 130.015; geodeticDatum: WGS84; **Identification:** identifiedBy: Haruka Tachi and Tadashi Ishikawa; dateIdentified: 2017; **Event:** samplingProtocol: none specified; eventDate: 2015-05-10; **Record Level:** institutionCode: ELTUA; collectionCode: IC; basisOfRecord: PreservedSpecimen
- **Type status:** Other material. **Occurrence:** catalogNumber: 2017-00021; recordedBy: Tatsuya Nozaki; individualCount: 1; sex: male; lifeStage: adult; **Taxon:** scientificName: Caystrusorientalis Zhang and Lin, 1985; namePublishedIn: 1985; kingdom: Animalia; phylum: Arthropoda; class: Insecta; order: Hemiptera; family: Pentatomidae; genus: Caystrus; specificEpithet: orientalis; scientificNameAuthorship: Zhang and Lin; **Location:** islandGroup: Amakusa Islands; island: Shimo-shima Island; country: Japan; stateProvince: Kumamoto; municipality: Amakusa City; locality: Oniki-machi, Oniki; decimalLatitude: 32.233; decimalLongitude: 130.015; geodeticDatum: WGS84; **Identification:** identifiedBy: Haruka Tachi and Tadashi Ishikawa; dateIdentified: 2017; **Event:** samplingProtocol: none specified; eventDate: 2015-10-17; **Record Level:** institutionCode: ELTUA; collectionCode: IC; basisOfRecord: PreservedSpecimen
- **Type status:** Other material. **Occurrence:** catalogNumber: 2017-00022; recordedBy: Tatsuya Nozaki; individualCount: 1; sex: male; lifeStage: adult; **Taxon:** scientificName: Caystrusorientalis Zhang and Lin, 1985; namePublishedIn: 1985; kingdom: Animalia; phylum: Arthropoda; class: Insecta; order: Hemiptera; family: Pentatomidae; genus: Caystrus; specificEpithet: orientalis; scientificNameAuthorship: Zhang and Lin; **Location:** islandGroup: Amakusa Islands; island: Shimo-shima Island; country: Japan; stateProvince: Kumamoto; municipality: Amakusa City; locality: Oniki-machi, Oniki; decimalLatitude: 32.233; decimalLongitude: 130.015; geodeticDatum: WGS84; **Identification:** identifiedBy: Haruka Tachi and Tadashi Ishikawa; dateIdentified: 2017; **Event:** samplingProtocol: none specified; eventDate: 2015-10-17; **Record Level:** institutionCode: ELTUA; collectionCode: IC; basisOfRecord: PreservedSpecimen
- **Type status:** Other material. **Occurrence:** catalogNumber: 2017-00023; recordedBy: Tadashi Ishikawa; individualCount: 1; sex: male; lifeStage: adult; **Taxon:** scientificName: Caystrusorientalis Zhang and Lin, 1985; namePublishedIn: 1985; kingdom: Animalia; phylum: Arthropoda; class: Insecta; order: Hemiptera; family: Pentatomidae; genus: Caystrus; specificEpithet: orientalis; scientificNameAuthorship: Zhang and Lin; **Location:** islandGroup: Ryukyu Islands; island: Ishigaki-jima Island; country: Japan; stateProvince: Okinawa; municipality: Ishigaki City; locality: near Maezato Dam; decimalLatitude: 24.42; decimalLongitude: 124.198; geodeticDatum: WGS84; **Identification:** identifiedBy: Haruka Tachi and Tadashi Ishikawa; dateIdentified: 2017; **Event:** samplingProtocol: none specified; eventDate: 2004-05-05; **Record Level:** institutionCode: ELTUA; collectionCode: IC; basisOfRecord: PreservedSpecimen
- **Type status:** Other material. **Occurrence:** catalogNumber: 2017-00024; recordedBy: Tatsuya Nozaki; individualCount: 1; sex: female; lifeStage: adult; **Taxon:** scientificName: Caystrusorientalis Zhang and Lin, 1985; namePublishedIn: 1985; kingdom: Animalia; phylum: Arthropoda; class: Insecta; order: Hemiptera; family: Pentatomidae; genus: Caystrus; specificEpithet: orientalis; scientificNameAuthorship: Zhang and Lin; **Location:** islandGroup: Amakusa Islands; island: Shimo-shima Island; country: Japan; stateProvince: Kumamoto; municipality: Amakusa City; locality: Oniki-machi, Oniki; decimalLatitude: 32.233; decimalLongitude: 130.015; geodeticDatum: WGS84; **Identification:** identifiedBy: Haruka Tachi and Tadashi Ishikawa; dateIdentified: 2017; **Event:** samplingProtocol: none specified; eventDate: 2015-10-17; **Record Level:** institutionCode: ELTUA; collectionCode: IC; basisOfRecord: PreservedSpecimen
- **Type status:** Other material. **Occurrence:** catalogNumber: 2017-00025; recordedBy: Tatsuya Nozaki; individualCount: 1; sex: female; lifeStage: adult; **Taxon:** scientificName: Caystrusorientalis Zhang and Lin, 1985; namePublishedIn: 1985; kingdom: Animalia; phylum: Arthropoda; class: Insecta; order: Hemiptera; family: Pentatomidae; genus: Caystrus; specificEpithet: orientalis; scientificNameAuthorship: Zhang and Lin; **Location:** islandGroup: Amakusa Islands; island: Shimo-shima Island; country: Japan; stateProvince: Kumamoto; municipality: Amakusa City; locality: Oniki-machi, Oniki; decimalLatitude: 32.233; decimalLongitude: 130.015; geodeticDatum: WGS84; **Identification:** identifiedBy: Haruka Tachi and Tadashi Ishikawa; dateIdentified: 2017; **Event:** samplingProtocol: none specified; eventDate: 2015-05-10; **Record Level:** institutionCode: ELTUA; collectionCode: IC; basisOfRecord: PreservedSpecimen
- **Type status:** Other material. **Occurrence:** catalogNumber: 2017-00026; recordedBy: Tatsuya Nozaki; individualCount: 1; sex: female; lifeStage: adult; **Taxon:** scientificName: Caystrusorientalis Zhang and Lin, 1985; namePublishedIn: 1985; kingdom: Animalia; phylum: Arthropoda; class: Insecta; order: Hemiptera; family: Pentatomidae; genus: Caystrus; specificEpithet: orientalis; scientificNameAuthorship: Zhang and Lin; **Location:** islandGroup: Amakusa Islands; island: Shimo-shima Island; country: Japan; stateProvince: Kumamoto; municipality: Amakusa City; locality: Oniki-machi, Oniki; decimalLatitude: 32.247; decimalLongitude: 129.987; geodeticDatum: WGS84; **Identification:** identifiedBy: Haruka Tachi and Tadashi Ishikawa; dateIdentified: 2017; **Event:** samplingProtocol: none specified; eventDate: 2015-05-18; **Record Level:** institutionCode: ELTUA; collectionCode: IC; basisOfRecord: PreservedSpecimen
- **Type status:** Other material. **Occurrence:** catalogNumber: 2017-00027; recordedBy: Tatsuya Nozaki; individualCount: 1; sex: female; lifeStage: adult; **Taxon:** scientificName: Caystrusorientalis Zhang and Lin, 1985; namePublishedIn: 1985; kingdom: Animalia; phylum: Arthropoda; class: Insecta; order: Hemiptera; family: Pentatomidae; genus: Caystrus; specificEpithet: orientalis; scientificNameAuthorship: Zhang and Lin; **Location:** country: Japan; stateProvince: Nagasaki; municipality: Nagasaki City; locality: Mieda; decimalLatitude: 32.82; decimalLongitude: 129.73; geodeticDatum: WGS84; **Identification:** identifiedBy: Haruka Tachi and Tadashi Ishikawa; dateIdentified: 2017; **Event:** samplingProtocol: none specified; eventDate: 2016-09-16; **Record Level:** institutionCode: ELTUA; collectionCode: IC; basisOfRecord: PreservedSpecimen
- **Type status:** Other material. **Occurrence:** catalogNumber: 2017-00028; recordedBy: Tatsuya Nozaki; individualCount: 1; sex: female; lifeStage: adult; **Taxon:** scientificName: Caystrusorientalis Zhang and Lin, 1985; namePublishedIn: 1985; kingdom: Animalia; phylum: Arthropoda; class: Insecta; order: Hemiptera; family: Pentatomidae; genus: Caystrus; specificEpithet: orientalis; scientificNameAuthorship: Zhang and Lin; **Location:** country: Japan; stateProvince: Nagasaki; municipality: Nagasaki City; locality: Mieda; decimalLatitude: 32.82; decimalLongitude: 129.73; geodeticDatum: WGS84; **Identification:** identifiedBy: Haruka Tachi and Tadashi Ishikawa; dateIdentified: 2017; **Event:** samplingProtocol: none specified; eventDate: 2016-03-21; **Record Level:** institutionCode: ELTUA; collectionCode: IC; basisOfRecord: PreservedSpecimen
- **Type status:** Other material. **Occurrence:** catalogNumber: 2017-00029; recordedBy: Tatsuya Nozaki; individualCount: 1; sex: female; lifeStage: adult; **Taxon:** scientificName: Caystrusorientalis Zhang and Lin, 1985; namePublishedIn: 1985; kingdom: Animalia; phylum: Arthropoda; class: Insecta; order: Hemiptera; family: Pentatomidae; genus: Caystrus; specificEpithet: orientalis; scientificNameAuthorship: Zhang and Lin; **Location:** country: Japan; stateProvince: Nagasaki; municipality: Nagasaki City; locality: Mieda; decimalLatitude: 32.82; decimalLongitude: 129.73; geodeticDatum: WGS84; **Identification:** identifiedBy: Haruka Tachi and Tadashi Ishikawa; dateIdentified: 2017; **Event:** samplingProtocol: none specified; eventDate: 2016-09-16; **Record Level:** institutionCode: ELTUA; collectionCode: IC; basisOfRecord: PreservedSpecimen
- **Type status:** Other material. **Occurrence:** catalogNumber: 2017-00030; recordedBy: Tatsuya Nozaki; individualCount: 1; sex: female; lifeStage: adult; **Taxon:** scientificName: Caystrusorientalis Zhang and Lin, 1985; namePublishedIn: 1985; kingdom: Animalia; phylum: Arthropoda; class: Insecta; order: Hemiptera; family: Pentatomidae; genus: Caystrus; specificEpithet: orientalis; scientificNameAuthorship: Zhang and Lin; **Location:** islandGroup: Amakusa Islands; island: Shimo-shima Island; country: Japan; stateProvince: Kumamoto; municipality: Amakusa City; locality: Oniki-machi, Oniki; decimalLatitude: 32.233; decimalLongitude: 130.015; geodeticDatum: WGS84; **Identification:** identifiedBy: Haruka Tachi and Tadashi Ishikawa; dateIdentified: 2017; **Event:** samplingProtocol: none specified; eventDate: 2015-10-17; **Record Level:** institutionCode: ELTUA; collectionCode: IC; basisOfRecord: PreservedSpecimen
- **Type status:** Other material. **Occurrence:** catalogNumber: 2017-00031; recordedBy: Tatsuya Nozaki; individualCount: 1; sex: female; lifeStage: adult; **Taxon:** scientificName: Caystrusorientalis Zhang and Lin, 1985; namePublishedIn: 1985; kingdom: Animalia; phylum: Arthropoda; class: Insecta; order: Hemiptera; family: Pentatomidae; genus: Caystrus; specificEpithet: orientalis; scientificNameAuthorship: Zhang and Lin; **Location:** islandGroup: Amakusa Islands; island: Shimo-shima Island; country: Japan; stateProvince: Kumamoto; municipality: Amakusa City; locality: Oniki-machi, Oniki; decimalLatitude: 32.233; decimalLongitude: 130.015; geodeticDatum: WGS84; **Identification:** identifiedBy: Haruka Tachi and Tadashi Ishikawa; dateIdentified: 2017; **Event:** samplingProtocol: none specified; eventDate: 2015-10-17; **Record Level:** institutionCode: ELTUA; collectionCode: IC; basisOfRecord: PreservedSpecimen
- **Type status:** Other material. **Occurrence:** catalogNumber: 2017-00032; recordedBy: Tadashi Ishikawa; individualCount: 1; sex: female; lifeStage: adult; **Taxon:** scientificName: Caystrusorientalis Zhang and Lin, 1985; namePublishedIn: 1985; kingdom: Animalia; phylum: Arthropoda; class: Insecta; order: Hemiptera; family: Pentatomidae; genus: Caystrus; specificEpithet: orientalis; scientificNameAuthorship: Zhang and Lin; **Location:** islandGroup: Ryukyu Islands; island: Ishigaki-jima Island; country: Japan; stateProvince: Okinawa; municipality: Ishigaki City; locality: near Maezato Dam; decimalLatitude: 24.42; decimalLongitude: 124.198; geodeticDatum: WGS84; **Identification:** identifiedBy: Haruka Tachi and Tadashi Ishikawa; dateIdentified: 2017; **Event:** samplingProtocol: none specified; eventDate: 2004-05-05; **Record Level:** institutionCode: ELTUA; collectionCode: IC; basisOfRecord: PreservedSpecimen
- **Type status:** Other material. **Occurrence:** catalogNumber: 2017-00033; recordedBy: Tatsuya Nozaki; individualCount: 1; sex: female; lifeStage: adult; **Taxon:** scientificName: Caystrusorientalis Zhang and Lin, 1985; namePublishedIn: 1985; kingdom: Animalia; phylum: Arthropoda; class: Insecta; order: Hemiptera; family: Pentatomidae; genus: Caystrus; specificEpithet: orientalis; scientificNameAuthorship: Zhang and Lin; **Location:** islandGroup: Amakusa Islands; island: Shimo-shima Island; country: Japan; stateProvince: Kumamoto; municipality: Amakusa City; locality: Oniki-machi, Oniki; decimalLatitude: 32.233; decimalLongitude: 130.015; geodeticDatum: WGS84; **Identification:** identifiedBy: Haruka Tachi and Tadashi Ishikawa; dateIdentified: 2017; **Event:** samplingProtocol: none specified; eventDate: 2015-10-17; **Record Level:** institutionCode: ELTUA; collectionCode: IC; basisOfRecord: PreservedSpecimen
- **Type status:** Other material. **Occurrence:** catalogNumber: 2017-00034; recordedBy: Kyohei Watanabe; individualCount: 1; sex: female; lifeStage: adult; **Taxon:** scientificName: Caystrusorientalis Zhang and Lin, 1985; namePublishedIn: 1985; kingdom: Animalia; phylum: Arthropoda; class: Insecta; order: Hemiptera; family: Pentatomidae; genus: Caystrus; specificEpithet: orientalis; scientificNameAuthorship: Zhang and Lin; **Location:** islandGroup: Ryukyu Islands; island: Iriomote-jim Island; country: Japan; stateProvince: Okinawa; municipality: Taketomi Town; locality: Otomi-rindo; decimalLatitude: 24.298; decimalLongitude: 123.863; geodeticDatum: WGS84; **Identification:** identifiedBy: Haruka Tachi and Tadashi Ishikawa; dateIdentified: 2017; **Event:** samplingProtocol: none specified; eventDate: 2006-09-11; **Record Level:** institutionCode: ELTUA; collectionCode: IC; basisOfRecord: PreservedSpecimen
- **Type status:** Other material. **Occurrence:** catalogNumber: 2017-00035; recordedBy: Reo Ito; individualCount: 1; sex: female; lifeStage: adult; **Taxon:** scientificName: Caystrusorientalis Zhang and Lin, 1985; namePublishedIn: 1985; kingdom: Animalia; phylum: Arthropoda; class: Insecta; order: Hemiptera; family: Pentatomidae; genus: Caystrus; specificEpithet: orientalis; scientificNameAuthorship: Zhang and Lin; **Location:** islandGroup: Ryukyu Islands; island: Okinawa-honto Island; country: Japan; stateProvince: Okinawa; municipality: Kunigami Village; locality: Ada, Fungawa Dam; decimalLatitude: 26.742; decimalLongitude: 128.279; geodeticDatum: WGS84; **Identification:** identifiedBy: Haruka Tachi and Tadashi Ishikawa; dateIdentified: 2017; **Event:** samplingProtocol: none specified; eventDate: 2011-05-25; **Record Level:** institutionCode: ELTUA; collectionCode: IC; basisOfRecord: PreservedSpecimen
- **Type status:** Other material. **Occurrence:** catalogNumber: 2017-00036; recordedBy: Haruka Tachi; individualCount: 1; sex: female; lifeStage: adult; **Taxon:** scientificName: Caystrusorientalis Zhang and Lin, 1985; namePublishedIn: 1985; kingdom: Animalia; phylum: Arthropoda; class: Insecta; order: Hemiptera; family: Pentatomidae; genus: Caystrus; specificEpithet: orientalis; scientificNameAuthorship: Zhang and Lin; **Location:** islandGroup: Ryukyu Islands; island: Yonaguni-jima Island; country: Japan; stateProvince: Okinawa; municipality: Yonaguni Town; locality: Mt. Kubura-dake; decimalLatitude: 24.454; decimalLongitude: 122.954; geodeticDatum: WGS84; **Identification:** identifiedBy: Haruka Tachi and Tadashi Ishikawa; dateIdentified: 2017; **Event:** samplingProtocol: none specified; eventDate: 2017-05-26; **Record Level:** institutionCode: ELTUA; collectionCode: IC; basisOfRecord: PreservedSpecimen
- **Type status:** Other material. **Occurrence:** catalogNumber: 2017-00037; recordedBy: Tadashi Ishikawa; individualCount: 1; sex: male; lifeStage: adult; **Taxon:** scientificName: Caystrusorientalis Zhang and Lin, 1985; namePublishedIn: 1985; kingdom: Animalia; phylum: Arthropoda; class: Insecta; order: Hemiptera; family: Pentatomidae; genus: Caystrus; specificEpithet: orientalis; scientificNameAuthorship: Zhang and Lin; **Location:** islandGroup: Ryukyu Islands; island: Ishigaki-jima Island; country: Japan; stateProvince: Okinawa; municipality: Ishigaki City; locality: near Maezato Dam; decimalLatitude: 24.42; decimalLongitude: 124.198; geodeticDatum: WGS84; **Identification:** identifiedBy: Haruka Tachi and Tadashi Ishikawa; dateIdentified: 2017; **Event:** samplingProtocol: none specified; eventDate: 2004-05-05; **Record Level:** institutionCode: ELTUA; collectionCode: IC; basisOfRecord: PreservedSpecimen
- **Type status:** Other material. **Occurrence:** catalogNumber: 2017-00038; recordedBy: Tadashi Ishikawa; individualCount: 1; sex: male; lifeStage: adult; **Taxon:** scientificName: Caystrusorientalis Zhang and Lin, 1985; namePublishedIn: 1985; kingdom: Animalia; phylum: Arthropoda; class: Insecta; order: Hemiptera; family: Pentatomidae; genus: Caystrus; specificEpithet: orientalis; scientificNameAuthorship: Zhang and Lin; **Location:** islandGroup: Ryukyu Islands; island: Ishigaki-jima Island; country: Japan; stateProvince: Okinawa; municipality: Ishigaki City; locality: near Maezato Dam; decimalLatitude: 24.42; decimalLongitude: 124.198; geodeticDatum: WGS84; **Identification:** identifiedBy: Haruka Tachi and Tadashi Ishikawa; dateIdentified: 2017; **Event:** samplingProtocol: none specified; eventDate: 2004-05-16; **Record Level:** institutionCode: ELTUA; collectionCode: IC; basisOfRecord: PreservedSpecimen
- **Type status:** Other material. **Occurrence:** catalogNumber: 2017-00039; recordedBy: Reo Ito; individualCount: 1; sex: female; lifeStage: adult; **Taxon:** scientificName: Caystrusorientalis Zhang and Lin, 1985; namePublishedIn: 1985; kingdom: Animalia; phylum: Arthropoda; class: Insecta; order: Hemiptera; family: Pentatomidae; genus: Caystrus; specificEpithet: orientalis; scientificNameAuthorship: Zhang and Lin; **Location:** islandGroup: Ryukyu Islands; island: Okinawa-honto Island; country: Japan; stateProvince: Okinawa; municipality: Kunigami Village; locality: Ada, Fungawa Dam; decimalLatitude: 26.742; decimalLongitude: 128.279; geodeticDatum: WGS84; **Identification:** identifiedBy: Haruka Tachi and Tadashi Ishikawa; dateIdentified: 2017; **Event:** samplingProtocol: none specified; eventDate: 2011-05-25; **Record Level:** institutionCode: ELTUA; collectionCode: IC; basisOfRecord: PreservedSpecimen
- **Type status:** Other material. **Occurrence:** catalogNumber: 2017-00040; recordedBy: Tadashi Ishikawa; individualCount: 1; sex: male; lifeStage: adult; **Taxon:** scientificName: Caystrusorientalis Zhang and Lin, 1985; namePublishedIn: 1985; kingdom: Animalia; phylum: Arthropoda; class: Insecta; order: Hemiptera; family: Pentatomidae; genus: Caystrus; specificEpithet: orientalis; scientificNameAuthorship: Zhang and Lin; **Location:** islandGroup: Ryukyu Islands; island: Ishigaki-jima Island; country: Japan; stateProvince: Okinawa; municipality: Ishigaki City; locality: near Maezato Dam; decimalLatitude: 24.42; decimalLongitude: 124.198; geodeticDatum: WGS84; **Identification:** identifiedBy: Haruka Tachi and Tadashi Ishikawa; dateIdentified: 2017; **Event:** samplingProtocol: none specified; eventDate: 2004-05-16; **Record Level:** institutionCode: ELTUA; collectionCode: IC; basisOfRecord: PreservedSpecimen
- **Type status:** Other material. **Occurrence:** catalogNumber: 2017-00041; recordedBy: Go Mashima; individualCount: 1; sex: female; lifeStage: adult; **Taxon:** scientificName: Caystrusorientalis Zhang and Lin, 1985; namePublishedIn: 1985; kingdom: Animalia; phylum: Arthropoda; class: Insecta; order: Hemiptera; family: Pentatomidae; genus: Caystrus; specificEpithet: orientalis; scientificNameAuthorship: Zhang and Lin; **Location:** islandGroup: Ryukyu Islands; island: Iriomote-jim Island; country: Japan; stateProvince: Okinawa; municipality: Taketomi Town; locality: Haeminaka; decimalLatitude: 24.292; decimalLongitude: 123.871; geodeticDatum: WGS84; **Identification:** identifiedBy: Haruka Tachi and Tadashi Ishikawa; dateIdentified: 2017; **Event:** samplingProtocol: none specified; eventDate: 2014-05-28; **Record Level:** institutionCode: ELTUA; collectionCode: IC; basisOfRecord: PreservedSpecimen
- **Type status:** Other material. **Occurrence:** catalogNumber: 2017-00042; recordedBy: Teruaki Ban; individualCount: 1; sex: female; lifeStage: adult; **Taxon:** scientificName: Caystrusorientalis Zhang and Lin, 1985; namePublishedIn: 1985; kingdom: Animalia; phylum: Arthropoda; class: Insecta; order: Hemiptera; family: Pentatomidae; genus: Caystrus; specificEpithet: orientalis; scientificNameAuthorship: Zhang and Lin; **Location:** islandGroup: Ryukyu Islands; island: Okinawa-honto Island; country: Japan; stateProvince: Okinawa; municipality: Ogimi Village; locality: Tsuha; decimalLatitude: 26.648; decimalLongitude: 128.092; geodeticDatum: WGS84; **Identification:** identifiedBy: Haruka Tachi and Tadashi Ishikawa; dateIdentified: 2017; **Event:** samplingProtocol: light trap; eventDate: 2007-05-23; **Record Level:** institutionCode: ELTUA; collectionCode: IC; basisOfRecord: PreservedSpecimen
- **Type status:** Other material. **Occurrence:** catalogNumber: 2017-00043; recordedBy: Tadashi Ishikawa; individualCount: 1; sex: female; lifeStage: adult; **Taxon:** scientificName: Caystrusorientalis Zhang and Lin, 1985; namePublishedIn: 1985; kingdom: Animalia; phylum: Arthropoda; class: Insecta; order: Hemiptera; family: Pentatomidae; genus: Caystrus; specificEpithet: orientalis; scientificNameAuthorship: Zhang and Lin; **Location:** islandGroup: Ryukyu Islands; island: Ishigaki-jima Island; country: Japan; stateProvince: Okinawa; municipality: Ishigaki City; locality: Sokobaru; decimalLatitude: 24.428; decimalLongitude: 124.212; geodeticDatum: WGS84; **Identification:** identifiedBy: Haruka Tachi and Tadashi Ishikawa; dateIdentified: 2017; **Event:** samplingProtocol: light trap; eventDate: 2000-05-24; **Record Level:** institutionCode: ELTUA; collectionCode: IC; basisOfRecord: PreservedSpecimen
- **Type status:** Other material. **Occurrence:** catalogNumber: 2017-00044; recordedBy: Kiyoshi Tanaka; individualCount: 1; sex: male; lifeStage: adult; **Taxon:** scientificName: Caystrusorientalis Zhang and Lin, 1985; namePublishedIn: 1985; kingdom: Animalia; phylum: Arthropoda; class: Insecta; order: Hemiptera; family: Pentatomidae; genus: Caystrus; specificEpithet: orientalis; scientificNameAuthorship: Zhang and Lin; **Location:** country: Japan; stateProvince: Nagasaki; municipality: Nagasaki City; locality: Mieda; decimalLatitude: 32.82; decimalLongitude: 129.73; geodeticDatum: WGS84; **Identification:** identifiedBy: Haruka Tachi and Tadashi Ishikawa; dateIdentified: 2017; **Event:** samplingProtocol: none specified; eventDate: 2016-05-10; **Record Level:** institutionCode: ELTUA; collectionCode: IC; basisOfRecord: PreservedSpecimen
- **Type status:** Other material. **Occurrence:** catalogNumber: 2017-00045; recordedBy: Kiyoshi Tanaka; individualCount: 1; sex: female; lifeStage: adult; **Taxon:** scientificName: Caystrusorientalis Zhang and Lin, 1985; namePublishedIn: 1985; kingdom: Animalia; phylum: Arthropoda; class: Insecta; order: Hemiptera; family: Pentatomidae; genus: Caystrus; specificEpithet: orientalis; scientificNameAuthorship: Zhang and Lin; **Location:** country: Japan; stateProvince: Nagasaki; municipality: Nagasaki City; locality: Mieda; decimalLatitude: 32.82; decimalLongitude: 129.73; geodeticDatum: WGS84; **Identification:** identifiedBy: Haruka Tachi and Tadashi Ishikawa; dateIdentified: 2017; **Event:** samplingProtocol: none specified; eventDate: 2015-06-05; **Record Level:** institutionCode: ELTUA; collectionCode: IC; basisOfRecord: PreservedSpecimen
- **Type status:** Other material. **Occurrence:** catalogNumber: 2017-00046; recordedBy: Kiyoshi Tanaka; individualCount: 1; sex: male; lifeStage: adult; **Taxon:** scientificName: Caystrusorientalis Zhang and Lin, 1985; namePublishedIn: 1985; kingdom: Animalia; phylum: Arthropoda; class: Insecta; order: Hemiptera; family: Pentatomidae; genus: Caystrus; specificEpithet: orientalis; scientificNameAuthorship: Zhang and Lin; **Location:** country: Japan; stateProvince: Nagasaki; municipality: Nagasaki City; locality: Mieda; decimalLatitude: 32.82; decimalLongitude: 129.73; geodeticDatum: WGS84; **Identification:** identifiedBy: Haruka Tachi and Tadashi Ishikawa; dateIdentified: 2017; **Event:** samplingProtocol: none specified; eventDate: 2015-06-05; **Record Level:** institutionCode: ELTUA; collectionCode: IC; basisOfRecord: PreservedSpecimen
- **Type status:** Other material. **Occurrence:** catalogNumber: 2017-00047; recordedBy: Tomohide Yasunaga; individualCount: 1; sex: male; lifeStage: adult; **Taxon:** scientificName: Caystrusorientalis Zhang and Lin, 1985; namePublishedIn: 1985; kingdom: Animalia; phylum: Arthropoda; class: Insecta; order: Hemiptera; family: Pentatomidae; genus: Caystrus; specificEpithet: orientalis; scientificNameAuthorship: Zhang and Lin; **Location:** country: Japan; stateProvince: Nagasaki; municipality: Nagasaki City; locality: Mieda; decimalLatitude: 32.82; decimalLongitude: 129.73; geodeticDatum: WGS84; **Identification:** identifiedBy: Haruka Tachi and Tadashi Ishikawa; dateIdentified: 2017; **Event:** samplingProtocol: none specified; eventDate: 2017-12-29; **Record Level:** institutionCode: ELTUA; collectionCode: IC; basisOfRecord: PreservedSpecimen

#### Diagnosis

*Caystrusorientalis* can be distinguished by a combination of the following characters: body 11.9–14.1 mm long (average 13.5 mm); body generally dark brown to blackish (Fig. 1); each mandibular plate not contiguous to another in front of clypeus (Fig. 1a, c); antennae brown to dark brown, with basal half of segment V pale (Fig. 1); mesal longitudinal stripe on pronotum and scutellum entirely unclear, often interrupted with blackish punctures (Fig. 1a, c); legs generally dark yellow to dark brown (Fig. 1b, d); venter of abdomen blackish, with connexivum yellowish to dark yellow, excluding blackish punctures (Fig. 1b, d); ventral rim of genital capsule (pygophore) medially notched in M-shape (Fig. 2a, b); M-shaped notch one-fifth as wide as genital capsule (Fig. 2b); apex of paramere triangular in caudal view, with dorsal lobe tapering and acute at apex and ventral lobe rounded at apex (Fig. 2c, d); apical receptacle of spermatheca provided with 2 processes, one directed upward or laterad, other directed downward (Fig. 2e).

#### Distribution

Japan: Kyushu (Nagasaki Prefecture, Kumamoto Prefecture), the Ryukyu Islands (Okinawa Island, Ishigaki Island, Iriomote Island, Yonaguni Island); China (Fujian Province, Jiangxi Province, Yunnan Province).

## Discussion

As mentioned above, two species of the genus *Caystrus*, *C.depressus* (Ellenrieder, 1862) and *C.obscurus* (Distant, 1901), were previously reported in Japan (Takara 1957, Ishikawa et al. 2012, Ishikawa 2016). Amongst the Japanese congeners, *C.orientalis* is separable from the other two species by a combination of the following characteristics: relatively large body, generally dark brown to blackish in colour; blackish abdomen venter, with a yellowish to dark yellow connexivum; ventral rim of the genital capsule with a medial M-shaped notch, which is one-fifth as wide as the genital capsule; triangular crown of paramere in caudal view, with the dorsal and ventral lobes acute and rounded at the apex, respectively; and apical receptacle of spermatheca with an upwardly or laterally directed process and a downwardly directed process.

**Figure 3.**
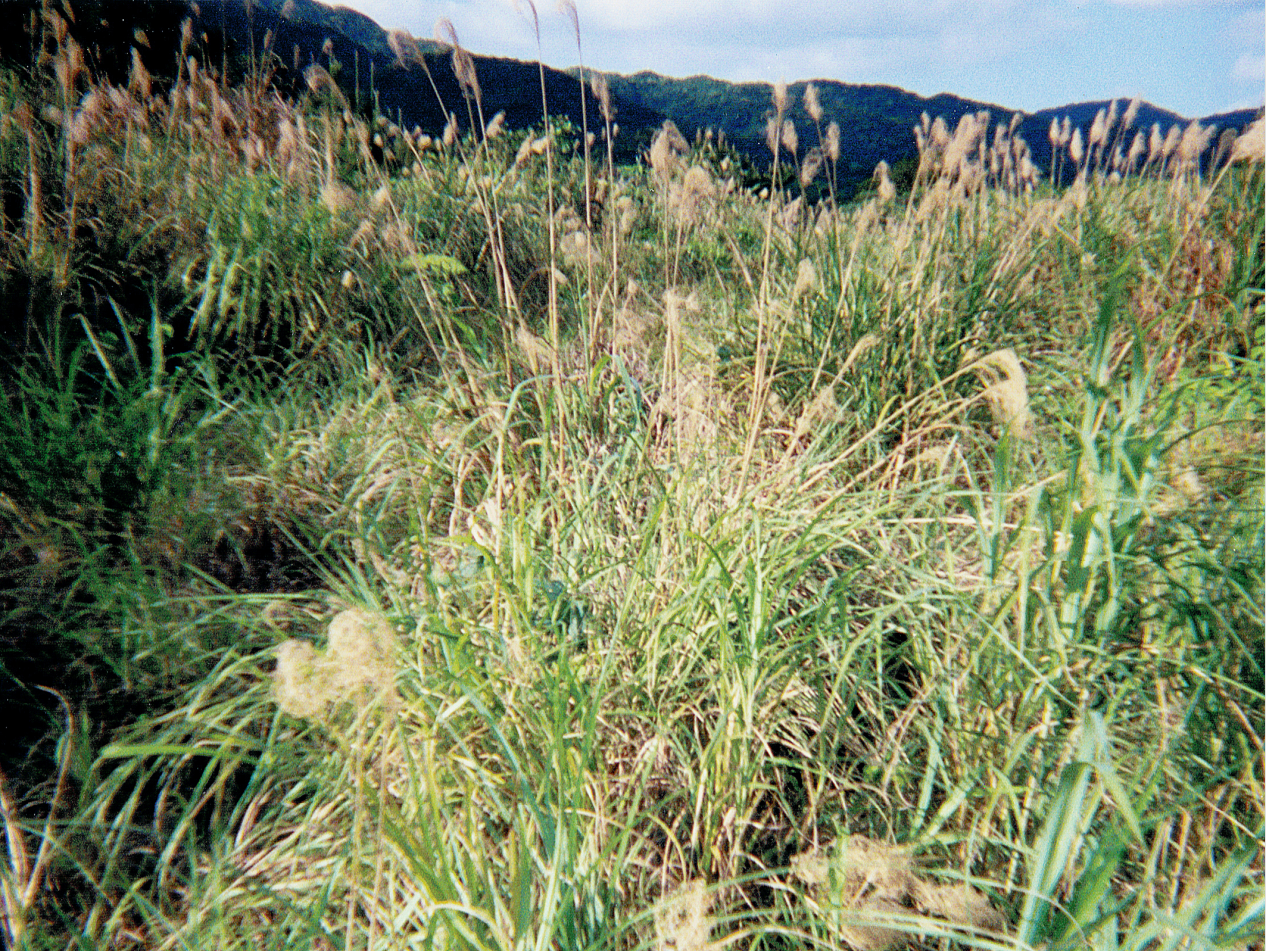
Habitat of *Caystrusorientalis* on Ishigaki Island, the Ryukyu Islands, Japan.

According to Nozaki et al. (2016), the adults and nymphs of *Caystrusorientalis* inhabit (or often coexist with) the stems or soft litter layers of the base of *Miscanthusfloridulus* (Labill.) Warb. (Poaceae) bushes in Kumamoto Prefecture, Kyushu, Japan. Similarly, we collected the Ryukyuan individuals of *C.orientalis* from the lower parts of *M.sinensis* Anderss bundles (Fig. 3). Therefore, this species appears to feed on *Miscanthus* grasses. Additionally, we found that the species was occasionally attracted to UV light at night, as reported in Nagasaki Prefecture (2001).

## Supplementary Material

XML Treatment for Caystrus orientalis

## Figures and Tables

**Figure 1a.**
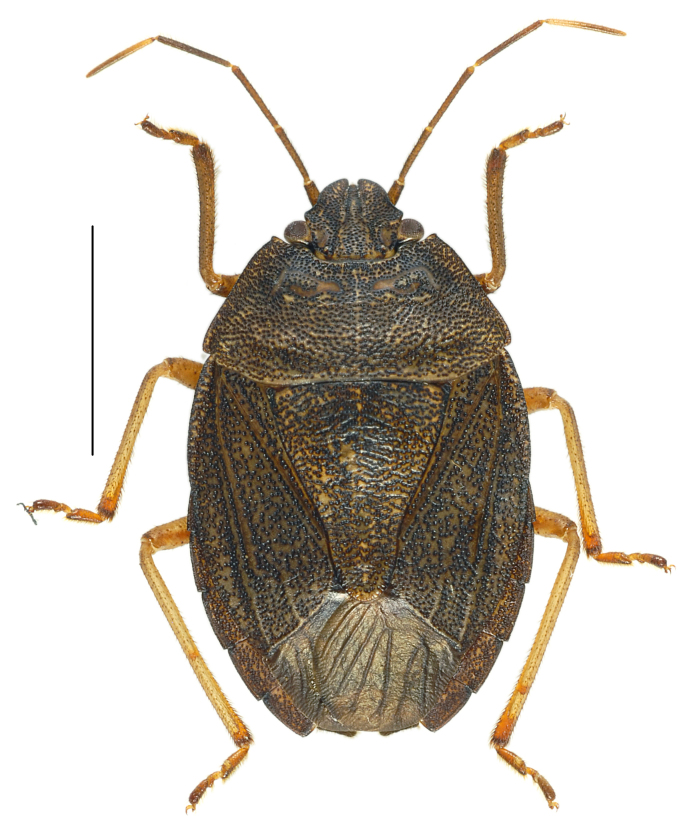
male, dorsal view.

**Figure 1b.**
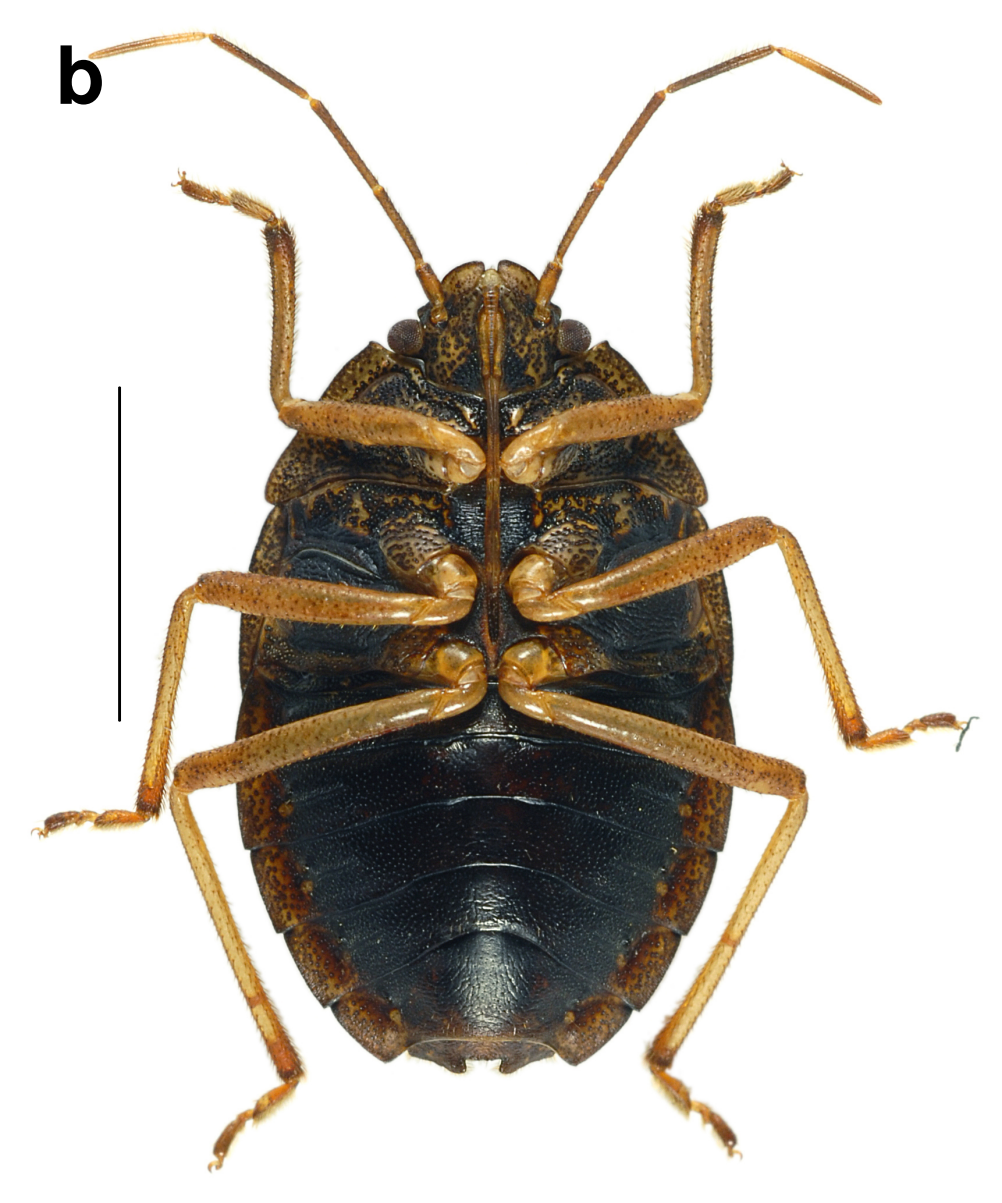
male, ventral view.

**Figure 1c.**
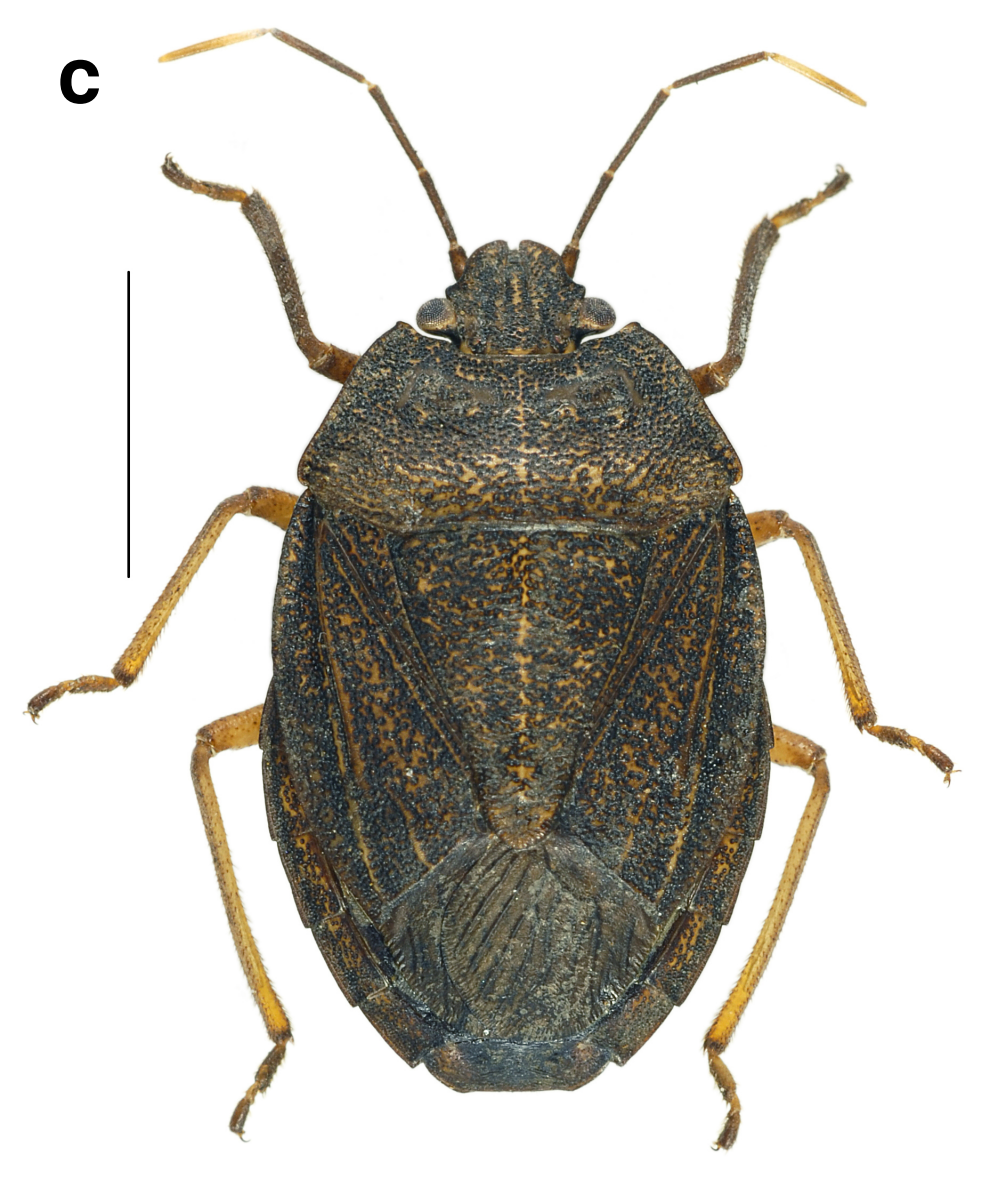
female, dorsal view.

**Figure 1d.**
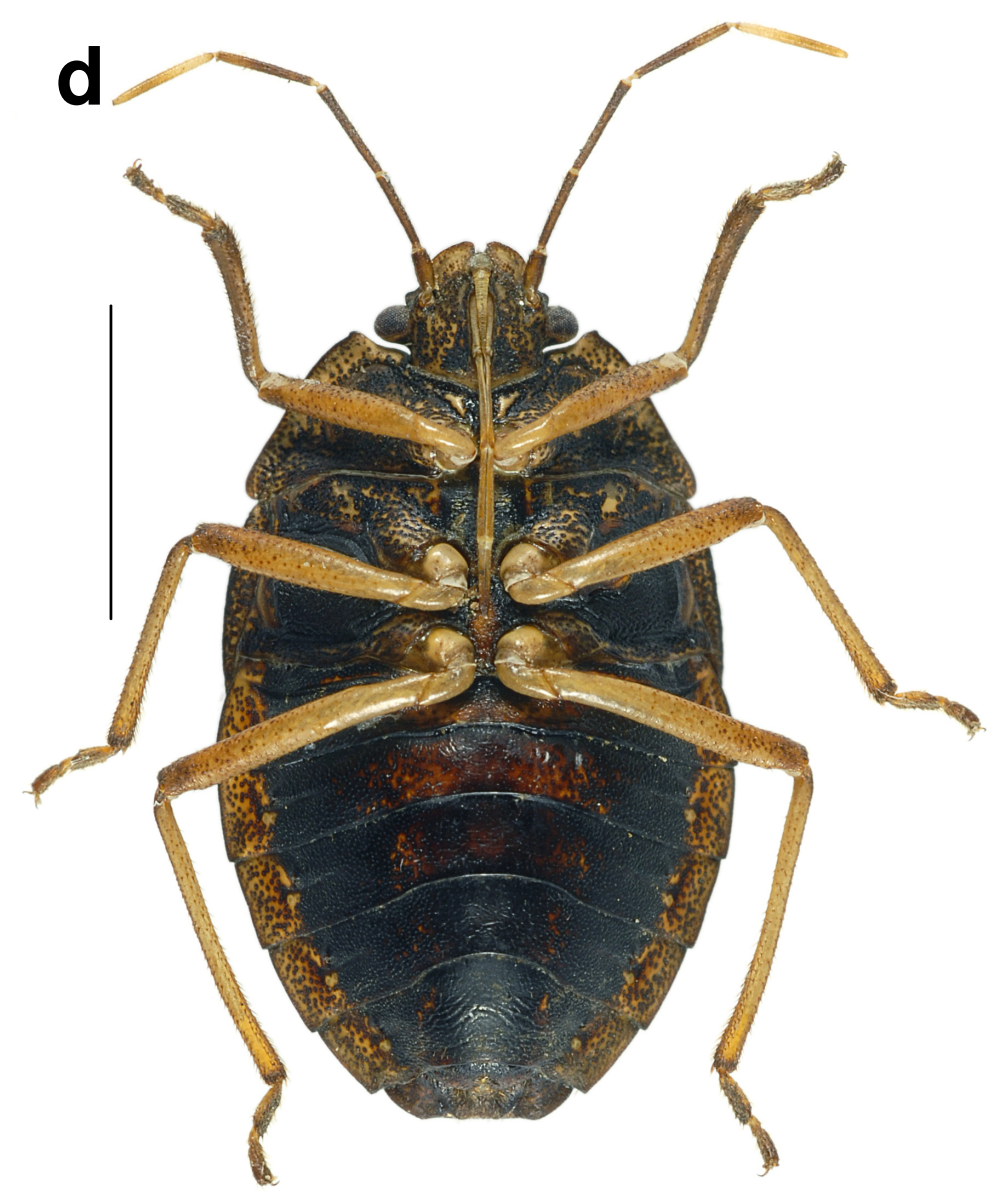
female, ventral view.

**Figure 2a.**
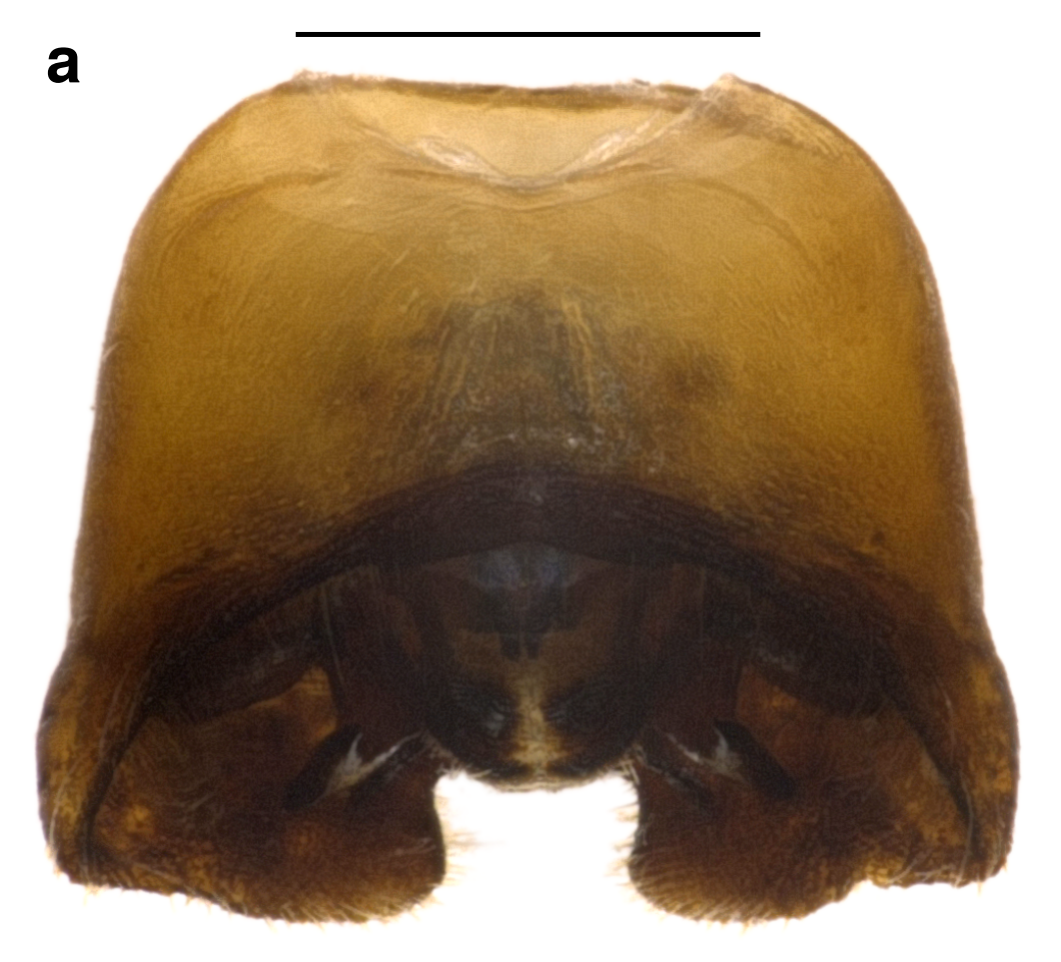
genital capsule (with parameres and abdominal segment X), dorsal view. Scale bar 1.0 mm.

**Figure 2b.**
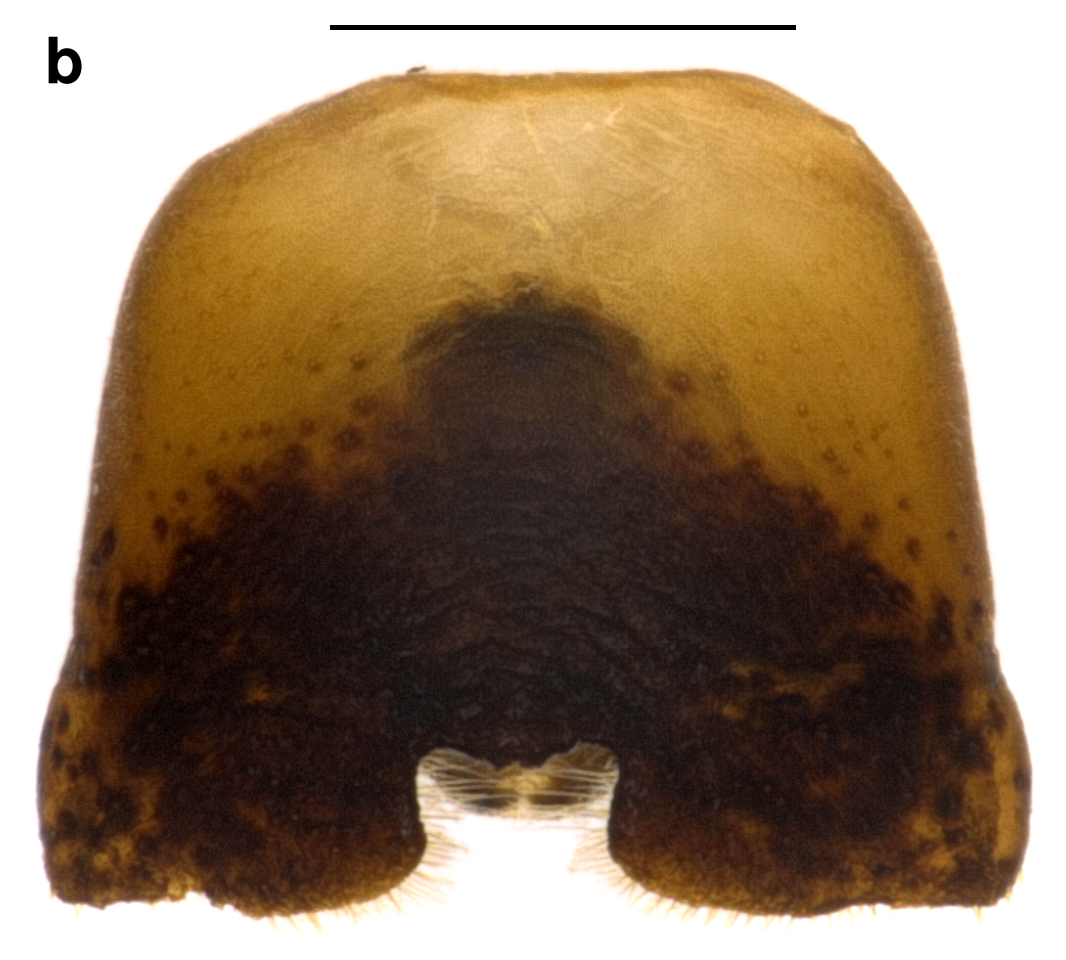
genital capsule (with parameres and abdominal segment X), ventral view. Scale bar 1.0 mm.

**Figure 2c.**
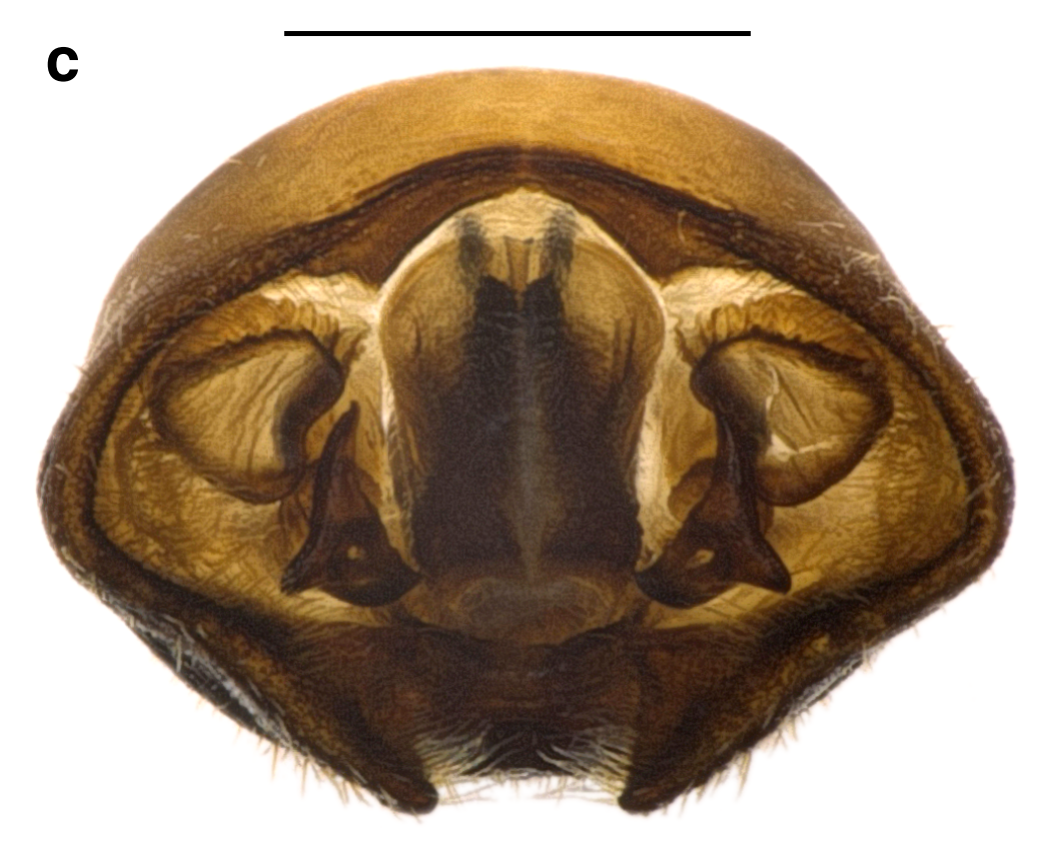
genital capsule (with parameres and abdominal segment X), caudal view. Scale bar 1.0 mm.

**Figure 2d.**
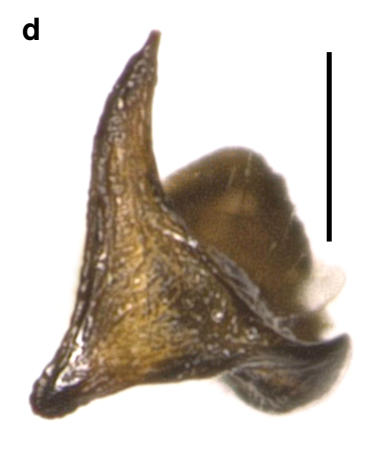
left paramere, caudal view. Scale bar 0.2 mm.

**Figure 2e.**
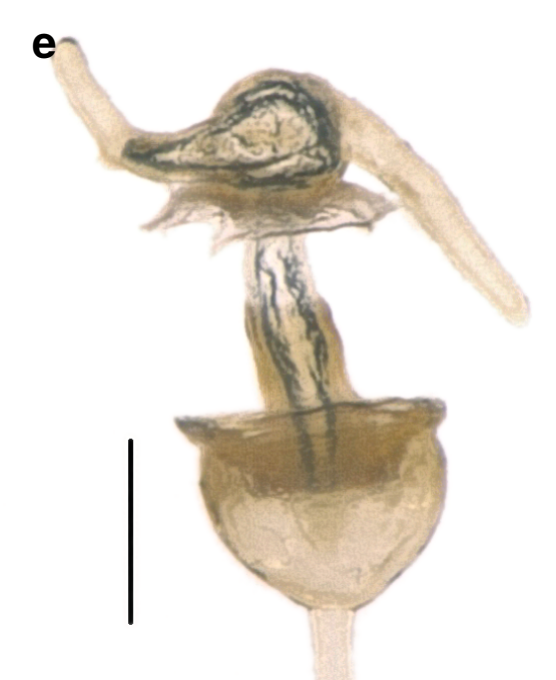
spermatheca. Scale bar 0.2 mm.
